# Heterochromatin Networks: Topology, Dynamics, and Function (a Working Hypothesis)

**DOI:** 10.3390/cells10071582

**Published:** 2021-06-23

**Authors:** Jekaterina Erenpreisa, Jekabs Krigerts, Kristine Salmina, Bogdan I. Gerashchenko, Talivaldis Freivalds, Reet Kurg, Ruth Winter, Matthias Krufczik, Pawel Zayakin, Michael Hausmann, Alessandro Giuliani

**Affiliations:** 1 Latvian Biomedicine Research and Study Centre, LV-1067 Riga, Latvia; jekabs.krigerts@gmail.com (J.K.); salmina.kristine@gmail.com (K.S.); pawel@biomed.lu.lv (P.Z.); 2 R.E. Kavetsky Institute of Experimental Pathology, Oncology and Radiobiology, National Academy of Sciences of Ukraine, 03022 Kyiv, Ukraine; biger63@yahoo.com; 3 Institute of Cardiology and Regenerative Medicine, University of Latvia, LV-1004 Riga, Latvia; freivald@latnet.lv; 4 Institute of Technology, University of Tartu, 50411 Tartu, Estonia; reet.kurg@ut.ee; 5 Kirchhoff Institute for Physics, Heidelberg University, 69120 Heidelberg, Germany; ruth.winter@kip.uni-heidelberg.de (R.W.); matthias@krufczik.de (M.K.); hausmann@kip.uni-heidelberg.de (M.H.); 6 Istituto Superiore di Sanita Environment and Health Department, 00161 Roma, Italy

**Keywords:** chromatin organization, heterochromatin, networks, positional information, scale-free oscillations, nucleolar boundary, transcriptional pulsing, cytoskeleton, physics of life

## Abstract

Open systems can only exist by self-organization as pulsing structures exchanging matter and energy with the outer world. This review is an attempt to reveal the organizational principles of the heterochromatin supra-intra-chromosomal network in terms of nonlinear thermodynamics. The accessibility of the linear information of the genetic code is regulated by constitutive heterochromatin (CHR) creating the positional information in a system of coordinates. These features include scale-free splitting-fusing of CHR with the boundary constraints of the nucleolus and nuclear envelope. The analysis of both the literature and our own data suggests a radial-concentric network as the main structural organization principle of CHR regulating transcriptional pulsing. The dynamic CHR network is likely created together with nucleolus-associated chromatin domains, while the alveoli of this network, including springy splicing speckles, are the pulsing transcription hubs. CHR contributes to this regulation due to the silencing position variegation effect, stickiness, and flexible rigidity determined by the positioning of nucleosomes. The whole system acts in concert with the elastic nuclear actomyosin network which also emerges by self-organization during the transcriptional pulsing process. We hypothesize that the the transcriptional pulsing, in turn, adjusts its frequency/amplitudes specified by topologically associating domains to the replication timing code that determines epigenetic differentiation memory.

*“Dissipative structures appear in fact as giant fluctuations, stabilized through matter and energy exchanges with the outer world”* 

[1]

## 1. Introduction

The DNA linear sequence code represents only the most basic principle of gene regulation. In 1944, Erwin Schrödinger (one of the founders of quantum mechanics and Physics Nobel Prize winner of 1933) published his famous lecture “What is life?” [2] and claimed the “hereditary molecule DNA” to be an aperiodic crystal that, as is typical for solid-state physics as such, uses spatial organization as one of the regulatory elements. Nowadays, the emerging picture of the cell nucleus indicates self-organizing feedback between function and structure of the genome [3,4,5] as the main driver to select, out of a two-meter DNA molecule compressed into the micrometer scale of a cell nucleus, the gene expression patterns necessary for life. “Selective unfolding” asks for phase separation, active and repulsive flows of chromatin compartments [6] flexibly acting like viscous liquids.

In general, as posed by Ilya Prigogine, any living dissipative system undergoing exchange of matter and energy with the environment is a subject of structured attenuated oscillations [1,7,8]. Thus, the 3D nuclear organization experiences transcriptional oscillations coordinating its function and scaling many orders of magnitude, including cell–cell interaction [9,10,11,12,13]. At a very coarse grain, we can imagine such oscillations as a biphasic interchange of chromatin compartments between active (compartment A) and inactive (compartment B) phases [14,15]. Recently, an intermediate compartment has been defined, rich with gene-possessing bivalent chromatin (i.e., having both repressive H3K27me3 and activating H3K4me3 histone modifications in the gene enhancer or promoter regions) and able to quickly shift the poised genes into active states and back [16]. This bivalent chromatin compartment characteristic of developmental genes is particularly enriched in cancer, determining its adaptive stress-related thermodynamics [17,18,19].

Although the dominating view in the last decade was that the A and B compartments behave as relatively independent self-organized crowded fluids, some recent data still point to the leading role of heterochromatin in genome maintenance, highlighting the attractions between heterochromatic regions as being central to phase separation of the active and inactive chromatin domains [20,21]. Recent experiments have shown that heterochromatin and bound HP1 protein undergo liquid-liquid phase separation, while euchromatin does not [22,23,24]. In addition, our studies of the optical features of DNA specifically stained with metachromatic absorption dye in intact cell nuclei revealed that ≥0.2 µm heterochromatin segments possess anisotropy (linear regularity), while euchromatin does not [25,26]. Moreover, the study of Strickfaden et al. [27] revealed that condensed chromatin exists in a solid-like state whose properties resist external forces and create an elastic gel providing a scaffold that supports liquid-liquid phase separation of chromatin binding proteins. The specific physical features of constitutive heterochromatin (CHR) composed of repeated short nucleotide sequences (satellite DNA), namely the regular structure, condensation, and positioning of nucleosomes, are conformed by a set of associated chromosomal proteins, including subcompartmentation by CTCF and cohesion, [23,28,29] which also depend evolutionarily on condensin II [30]. The particular role of chromatin modulators of the long noncoding RNA transcribed from heterochromatin is also being studied [31].

The three main properties of CHR—the position variegation effect, stickiness [32,33], and flexible rigidity [23,29,34]—are important for its network-related genome regulation and the prerequisite for network formation. These properties of CHR are consistent with the earlier ideas that heterochromatin topology, in terms of the sum of its position-cis-silencing effects on transcription, can provide the 3D “genome active space” in addition to the DNA gene-coding function, “the morphogenic function” [35]—a source of positional information necessary for the whole genome regulation [8,36,37,38,39,40,41,42]. Accordingly, Singh and Huskisson proposed that the physical features of heterochromatin may confer the specificity to provide a gene with “a chromosomal address” [43].

In our recent publication [44], we were able to link the timing of structural bursting of pericentromere-associated domains (PADs) with the large-scale activation of gene expression occurring by critical self-organization in cancer cells in the differentiation commitment model coinciding with biphasic activation of bivalent stress-response genes of the FOS family. Loss of the heterochromatin silencing threshold, which depends mostly on histone H3 lysine 9 methyltransferase SU(VAR)3-9 and creates a core memory system through the binding protein HP1a [45] by splitting PAD clusters leading to the documented massive unfolding of euchromatin, was suggested as the first step for genome repatterning. The discovered basic features of PADs, converting the same amount of constitutive heterochromatin by fusing-splitting within the constraint of cell nucleus volume, suggests their involvement in the supra-chromosomal network as one of the most promising structural candidates supporting chromatin driving gradients and transcriptional pulsing by bursting synchronization [46].

The mesoscopic approach to the description of complex systems is guided by unveiling the presence of “organization principles”. Its main peculiarity is the independence from the detailed knowledge of the basic bricks of the system and the emergence of relevant features from the correlation structure of the studied phenomenon, where “all the entities can be considered as networks of interacting parts” [47]. Here, we will follow this approach to individuate (besides a detailed molecular-level mechanistic approach) some basic pillars of the supra-chromosome wiring structure.

In this article, we will review the state of several relevant aspects of cell nucleus research and hypotheses for a “heterochromatin code” from the perspective of Schrödinger’s aperiodic crystal and a general concept of positional information as it was formulated for cellular morphogenesis by Leo Wolpert [48,49]. According to this concept “the cells acquire positional identities as in a coordinate system, and then interpret their positions to give rise to spatial patterns”. We will suggest that topology induces patterns that control functions, while functioning induces pattern pulsations followed by feedback oscillations. To support the general aspects in detail, we will present some data on heterochromatin network visualization and features obtained by image analysis and their functional-structural changes associated with developmental and transcription shifts available from the literature and our own data.

## 2. Visualization of the Supra-Chromosomal Networks

Roughly, we discriminate two main complementary-opposite components of the supra-chromosomal functional network: The “knots”, i.e., pericentromere-associated domains (PADs) formed by densely packed centromere aggregates, and the transcription hubs, i.e., transcription factories including splicing speckles for synthesis and processing of mRNA, which converge the loops of transcriptionally active chromatin domains [4,50,51]. In this bipartite segregation, the second component also includes nucleoli, the most abundant transcription factories for rRNA. Both compartments should form an integrated system composed of the PAD-knotted supra-chromosomal network and its alveoli, respectively.

In order to reveal the supra-chromosomal network knotted by CHR, image analysis of in situ microscopic cell nuclei was applied: (1) By labeling it with epigenetic histone mark H3K9me3 alone or with CENPA (using epifluorescent, confocal, and super-resolution localization microscopy), without and after suppression of RNA synthesis; (2) by affinity staining for CHR by absorption dye toluidine blue (using bright-field microscopy); (3) by mathematical skeletonization of denser nuclear areas and overlay of the most dense CHR clusters in cell nuclei with specifically stained DNA in cell imprints, studied in the course of developmental acceleration and cessation of transcription; (4) by extracting RNA from cell nuclei with nucleic acid specifically stained by acridine orange (AO), without and after suppression of RNA synthesis; (5) by electron microscopy of cell nuclei treated with heparin, short bleomycin, or DNAse I—cutting, solubilizing, and extracting the active chromatin on isolated whole nuclear mounts treated on EM supports and in situ for ultra-thin sections in cell cultures.

**Figure 1:**
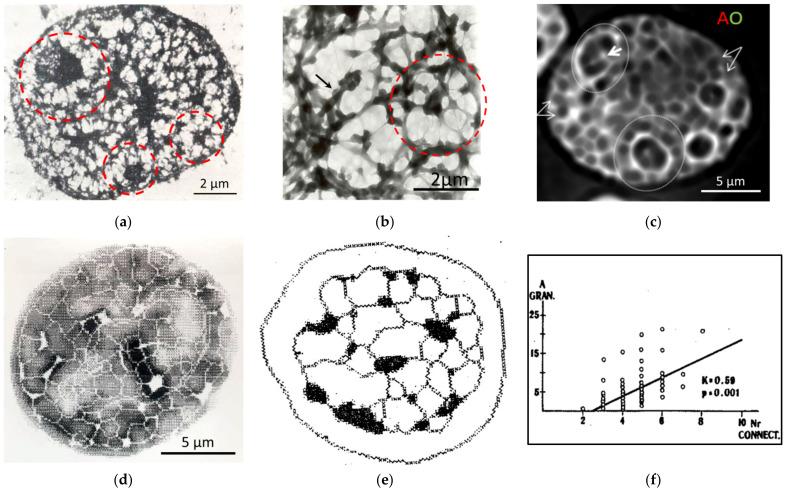
Visualization of the heterochromatin network in interphase cells: (**a,b**) Electron microscopic images of isolated rat thymocyte nuclei treated with 0.1% heparin/HBSS on supports; (**a**) 1 min; (**b**) 25 min (the nucleus fragment) by the method from [55]. PF fixation, uranyl acetate contrasting, tungsten oxide shadowing. (**c**) Human MCF7 cell, AO-DNA (after RNAse) fluorescent staining, deconvolution. (**a**–**c**) Large rosettes around nucleoli are encircled, heterochromatin clumps and “foots” inside them are arrowed, double alveoli are marked by double arrows. (**d**) Tissue imprint of chicken embryonic chondrocyte stained stoichiometrically for DNA; the method of image processing is described in [56]. (**e**) Mathematically skeletonized network of a DNA-stained chicken mesenchymal cell with overlaid dense chromocenters. (**f**) The relationship between the area of the dense chromocenters and the number of their network branches determined on chicken image-processed nuclei. Figure 1a,b republished from [52]. Figure 1d–f republished from [57].

**Figure 2:**
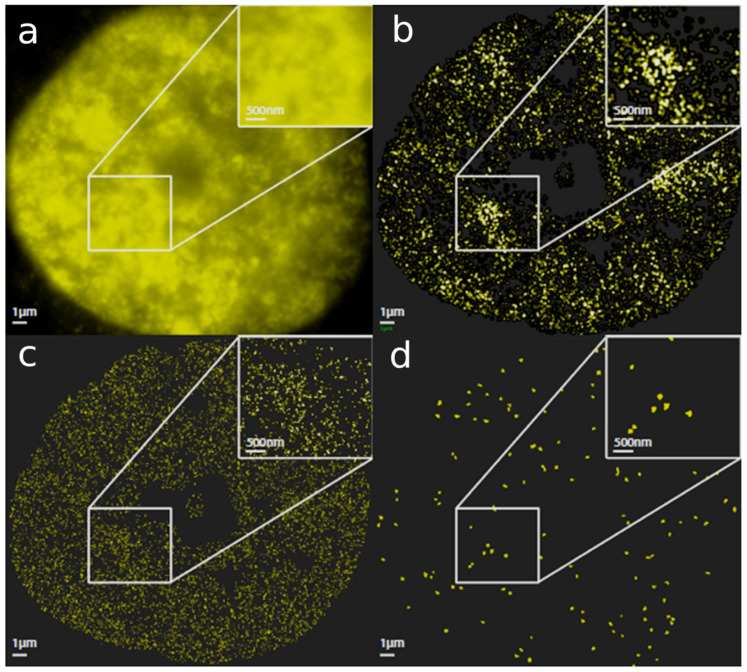
Resolution of the heterochromatin network reconstructed from loci data of fluorescent molecules of an H3K9me3-stained breast cancer SKBr3 nucleus in a localization microscope. The images of an SkBr3 (breast cancer cell line) cell nucleus show: (**a**) A fluorescence wide-field image after heterochromatin staining with specific antibodies against H3K9me3 methylation sites. The inset shows a magnification of a region around small alveoli. (**b**) The same cell nucleus reconstructed from the localization data of the single fluorescence molecules as a density map. This means that point intensity relates to the number of neighbors. Due to soft focus filters, details of molecular arrangements are lost, but high (knots) and low (alveoli) density structures can be easily detected. Such image reconstructions based on density histograms are similar to conventional microscope images and allow a comparison to other techniques in many cases. The insert indicates that the shapes obtained by standard microscopy can be resolved into heterochromatin arrangements of different shape and molecular density. (**c**) Full pointillist localization image of all fluorescent molecules detecting H3K9me3 sites. Such images are obtained from data sets that are the basis for all quantitative structure and topology analyses using mathematical algorithms of statistics, geometry, and topology. The inset qualitatively indicates that the high-density knots revealed a structured folding. (**d**) Cluster image as a result of mathematical operations on the localization data set (visualized in (**c**)). The image only shows the areas that met a certain cluster parameter. The insert indicates that the dense heterochromatin regions consisted of clusters embedded in heterochromatin structures shown in (**c**).

Visual inspection of the images revealed several general features of thus highlighted heterochromatin networks: On whole mounts contrasted for electron microscopy after heparin extraction (leaving ~25% of DNA content), the networks confined by arrays of ~1.12 µm-thick knotty chromatin strands were composed of smaller and larger alveoli (~2 µm on average). The larger ones generally embraced the apparent nucleoli and had a rosette-like substructure (encircled in Figure 1a,b) with a heterochromatin “clump” (this expression refers to the first visual impression) in their center, often seen entering from the side on a “foot” (Figure 1b,c) [52,53]. In the well-resolving staining of DNA by AO in intact MCF7 cells, with an average diameter of the alveoli of 1.87 ± 0.74 µm, the smaller alveoli were often observed in splitting doublets—the sign of symmetry break. They also tended to fuse in tandems (Figure 1c). These celled channels likely correspond to the “interchromatin spaces” previously described as harboring spliceosomes and products for export routes of mRNA towards nuclear pores [54]. After the suppression of RNA synthesis, the AO-defined average diameter of alveoli increased up to 2.12 ± 0.67 µm (*p* < 0.05). The chromocenters, when discriminated by our approach, either as the most optically dense DNA-specifically stained granules (Figure 1d,e) or as H3K9me3-stained densely clustered regions (Figure 2b), were most often found at knots or on the marginal ribs of a network (Figure 1e). The knotting parameter was determined for one of the studied models of chicken embryonal chondrocyte nuclei (with the diameter of the network alveoli ranging from 1.5 to 1.95 µm). It corresponded to the relationship between the area of chromocenters and the number of their branches reaching a statistically significant Pearson correlation of r = 0.59 (*p* < 0.01) (Figure 1f).

Using antibody labelling against heterochromatin methylation sites H3K9me3, fluorescence imaging (Figure 2a) can be downscaled to super-resolution single-molecule localization microscopy [58] (Figure 2b–d). This light-microscopic technique allows to determine the coordinates of single fluorescent molecules. From these data, artificial images can be prepared that contain not only points of molecular loci but also the results of mathematical operations on the recorded data sets, so that relevant substructures can be highlighted. Next, neighbor density images (Figure 2b) highlight the formation of highly dense CHR knots, corresponding very well to the results obtained by DNA density or electron-density measurements (after euchromatin extraction) with two other methods (Figure 1b,e). The mean diameter of the “alveoli” in SkBr3 cells was 1.7 µm. Deeper insight into folding can also be quantitatively obtained using the loci data set as visualized in Figure 2c. The application of cluster algorithms [59] defines the core clusters within the heterochromatin areas (Figure 2d). The results shown in Figure 2c,d indicate that the highly dense regions of CHR visualized by standard microscopy included lower-level subdomains. These may give them enough flexibility for reorganization that employs bending, splitting, fusing, and oscillation phenomena. This conclusion, obtained from super-resolution microscopy, is supported by investigations of nucleosome topology in mouse embryonic stem cells [29].

The networks visualized by five different labelling methods in different cell types and species (chicken, rat, human) showed similar sizes of the alveoli in all samples (Table 1, see also Section 3). This invariance points to the existence of shared organization principles of heterochromatin network and at the same time suggests the adequacy of the applied visualization methods.

## 3. Dynamics of the Heterochromatin Network: Boundaries and Radial Gradient

**Figure 3:**
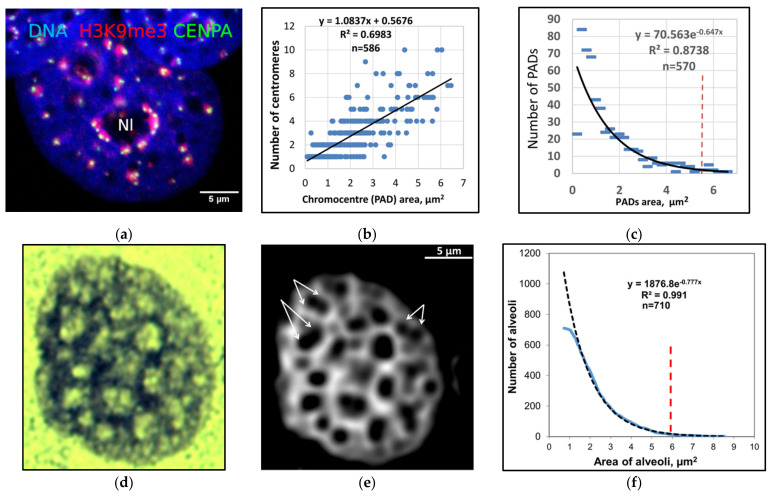
The features of PADs and their network show a scale-free distribution in control MCF7 cells. (**a**,**b**) H3K9me3-positive PADs are clustered by centromeres, Nl—nucleolus; (**c**) the area and number of individual PADs clustered by centromeres and the negative exponential relationship between the area and number of individual PADs, with the dashed boundary of the largest likely static PADs; (**d**) preferential staining of heterochromatin by toluidine blue (method from [61]); (**e**) the filtered image of the same nucleus revealed the heterochromatin network with typical double alveoli (double-arrowed); (**f**) the negative exponential relationship between the area and the number of individual network alveoli with a set dashed boundary for the largest ones; (**a**–**c**) republished from [44].

In MCF7 cells, we observed a positive correlation between H3K9me3-stained PADs and centromere numbers (Figure 3a,b) along with the scale-free distribution governing PADs number and size relation (Figure 3c) [44]. This distribution suggests the presence of fusing/splitting PADs, reorganizing an invariant amount of material, allowing a rapid (“all-or-none”) change [60]. The original method of affinity staining of heterochromatin with toluidine blue (including its RNA component) [61] was also applied on the same MCF7 serum-starved cells (Figure 3d). We used this simple staining of heterochromatin to contrast the alveolar network in MCF7 cells, employing interactive image filtering algorithms (using Image-Pro Plus software). The alveolar network obtained by such means seemed fair when visually compared with that in the original images (Figure 3d,e). The average size of the network alveoli was 1.71 ± 0.43 µm. Double alveoli were also evident, like in AO-staining (Figure 3e, double arrows). The size and the number of alveoli showed a clear negative exponential relationship (Figure 3f), which was very similar to the size/frequency distribution of PADs (Figure 3c). This finding suggests that both PAD´s reorganization and modulation of alveolar networks may be involved in the same scale-free physical process of pulsing [46].

Raymond Noble and colleagues, discussing the complexity of biological systems, recently posed: “Biological relativity requires circular causality but not a symmetry of causation: so, where, what, and when are the boundaries?” [62]. Another biophysical aspect of the same causation was set in the lab of Kenichi Yoshikawa [63], who showed that a lipid droplet undergoes regular rhythmic motion under appropriately designed boundary conditions, whereas it exhibits random motion in an isotropic environment. Therefore, the presence of boundaries (in a structure with finite size) can provoke ordered motion of heterochromatin domains.

In conventional eukaryotic interphase cells, heterochromatin segregates towards the perinucleolar and inner nuclear envelope surfaces [64,65,66,67], revealing these boundaries. Our measurements in individual poorly differentiated MCF7 breast cancer cells showed that the dynamics of the PAD network were restricted by the relatively static spatial determinant of a few large clusters of nucleolar organizers (NORs) at the central nucleolus [44]. A similar constraint was observed for large alveoli of the heterochromatin network in these cells (Figure 3c,f, on the right from the red dashed line), suggesting its pulsing from this boundary. Similarly, the nucleolar determinant with large alveoli was also revealed and characterized in the unique model of a chicken embryonic femur growth plate [38,57,68].

**Figure 4:**
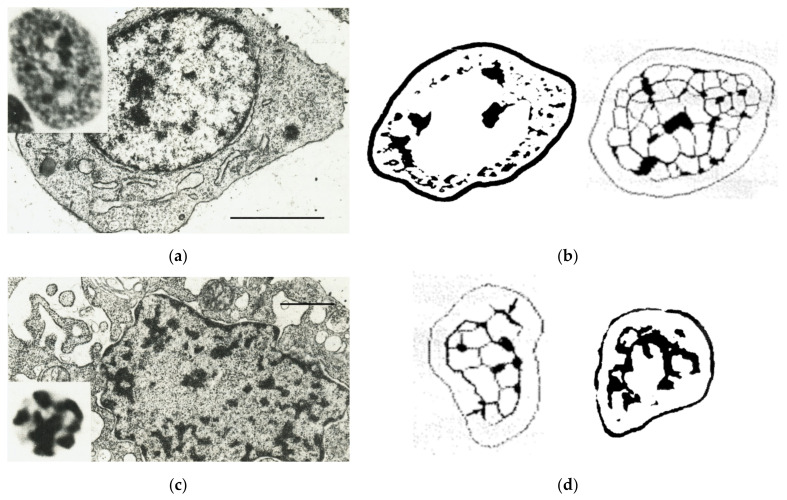

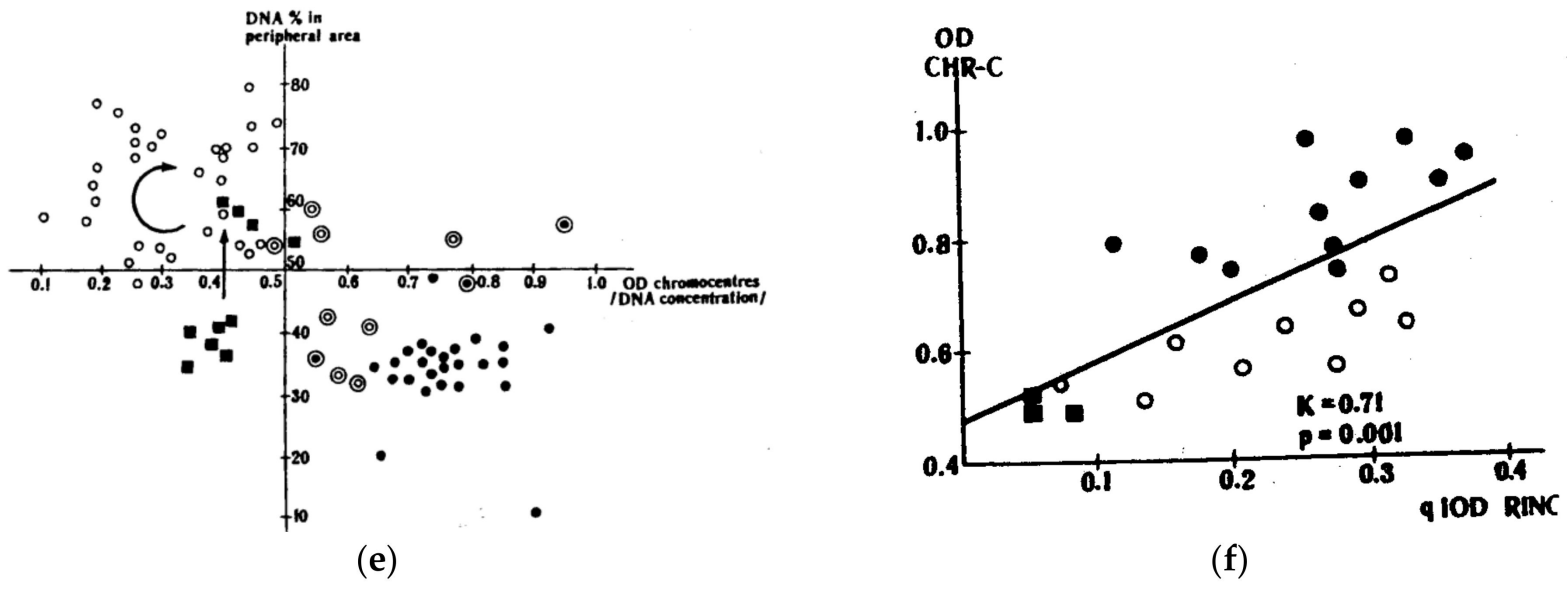
Chromatin changing in a chicken embryonic femur growth plate during transcriptional activation (**a**,**b**) and suppression (**c**,**d**) in the transient tissue—hypertrophic chondrocytes of a 14-day chicken embryo femur growth plate; (**e**,**f**) the results of morphometry showing the relationship between DNA concentration (OD) in dynamic chromocenters depending on the stage and vectorial position between the interactively discriminated perinucleolar heterochromatin shell (qIOD ring) and nuclear border in the active (light circles) and ageing (filled circles) chondrocytes; squares—mesenchymal precursors; double circles—differentiated, transient to ageing cells. (**f**) The concerted condensation of small chromocenters (Y-axis) and DNA accumulation in the perinucleolar ring formed by NORs and attached chromocenters (X-axis) in ageing cells (filled rings), and much weaker dependence on NORs in transcriptionally active chondrocytes (light circles). The mesenchymal precursors (squares in (**e**,**f**)) are rather inert to these forces. (**a**,**c**) Scale bars = 5 µm. Republished from [38,57,68].

In this system, presented in Figure 4, in the transient tissue—hypertrophic chondrocytes with highly accelerated transcription and loosely packed chromatin (Figure 4a, the pattern of DNA staining is shown on insert)—the emergence of peripheral small heterochromatin granules (up to 0.22 µm in diameter), moving radially from the perinucleolar heterochromatin shell and setting small network alveoli there, was found [38,57]. The typical processed images are shown in Figure 4b, and the quantified radial distribution of low DNA density small chromocenters can be seen in Figure 4e (upper left quadrant, light circles). On the contrary, when ceasing transcription with still intensive secretion activity, these cells underwent the programmed terminal differentiation with ageing in G2-phase and condensation of heterochromatin (Figure 4c); the morphometric analysis is exemplified in Figure 4d, and quantification in Figure 4e (lower right quadrant, filled circles for ageing chondrocytes and double circles for transient forms). It revealed that at this phase, small peripheral heterochromatin granules from two neighboring network knots fused as two rods into the dense marginal rib of the breaking alveoli (Figure 4d, left, arrows). It was occurring along with retraction of the nuclear envelope and shrinking the peripheral nuclear space (Figure 4e, lower right quadrant). Finally, the linearized dense segments of the broken alveoli were intruded into chromosome arms, and the latter contracted back towards the perinucleolar heterochromatin shell (Figure 4d, right, and Figure 4f) absorbing up to 30% of euchromatin DNA in its large chromocenters [68]. These ageing cells thus underwent premature mitosis and subsequently died by apoptosis (Figure 4c, insert) [69].

The radial movement vector of the dynamic, small chromocenters and the network, first creating and expanding its small alveoli and then flopping and contracting them towards the perinucleolar boundary during activation and irreversible cessation of transcription, correspondingly decreasing and increasing DNA concentration in them, is seen well in the graphs on Figure 4e,f. Finally, both nuclear shells collapse in shrunk terminal apoptotic cells. In accord with these observations, the radial expansion and partial intermingling of chromosome territories were found in PHA-activated lymphocytes [70], while nucleolus association with chromatin domains was reported in ageing IMR90 cells increasing with a decrease of transcription [71]. The formation of heterochromatin in the nucleolus at rRNA genes was shown to promote heterochromatinization (mediated by long noncoding RNA) of the rest of the nuclear genome; this process is also required to exit from pluripotency [72,73].

Similar contraction of CHR around/inside nucleoli was also observed at the initial suppression of rRNA synthesis by actinomycin D (AcD) [74,75]; this phenomenon is discussed below in more detail. The two boundaries of chromatin mobility at the nucleolar and nuclear shells were also confirmed in human cells by live-cell tracking of GFP-tagged genomic regions [76].

Thus, the concentric constraints of the nucleolus and nuclear envelope form the radial gradient of the heterochromatin network mobility vectors which coincide with the generally radial position of chromosome territories in interphase cell nuclei [77] known since its discovery by Boveri in 1988, cited from [78] and Rabl [79]. They are preformed around a central nucleolus in the late S-G2-phase [80] and seen in mitosis and meiosis. The role of nucleolar and nuclear envelope determinants for cell nucleus organization by heterochromatin was established nearly 50 years ago [81] and detailed by more recent studies [21,65,82].

The radial vector represents the shortest path of network signal transduction for its main function—linking DNA transcription to RNA translation between the cell nucleus and cytoplasm in oscillating cycles, as studied in a model system [83]. The complex oscillating networks consist of excitable nodes which appear in directly interacting pairs. In each pair, one node drives the other by a bidirectional link between the two, while the other excites the whole network. The authors found that most oscillations had self-organized structures with an extremely small number of center nodes. The two principles for pattern formation in a given network oscillation are proposed in this study: (1) Waves propagate forward from center nodes to the whole network along the shortest paths. (2) The shortest loops, which pass through both the center nodes and their driver nodes, play the role of oscillation sources and dominate the oscillation behavior. It seems that these results may apply to the observed properties of the nuclear intra-supra-chromosomal networks, at least as a working hypothesis. 

The clusters of constitutive heterochromatin are composed of multiple reiterated satellite DNA sequences. They may serve as “capacitors” for the induction of locally different electric potentials for chromatin and DNA [37,84,85,86], changing transcriptional accessibility and thus providing transduction of the oscillatory signal.

## 4. Phase Transitions of the CHR Network and Its Determinants in Early Embryo Development

**Figure 5:**
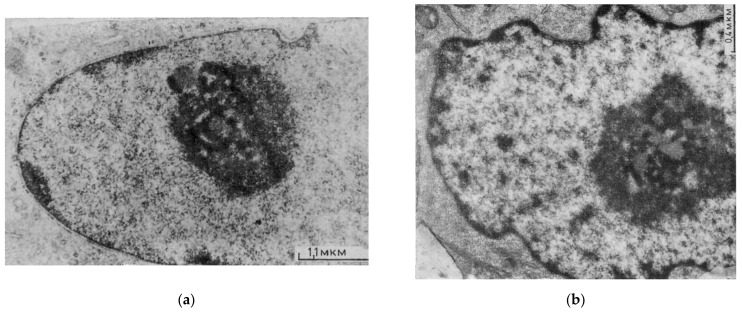

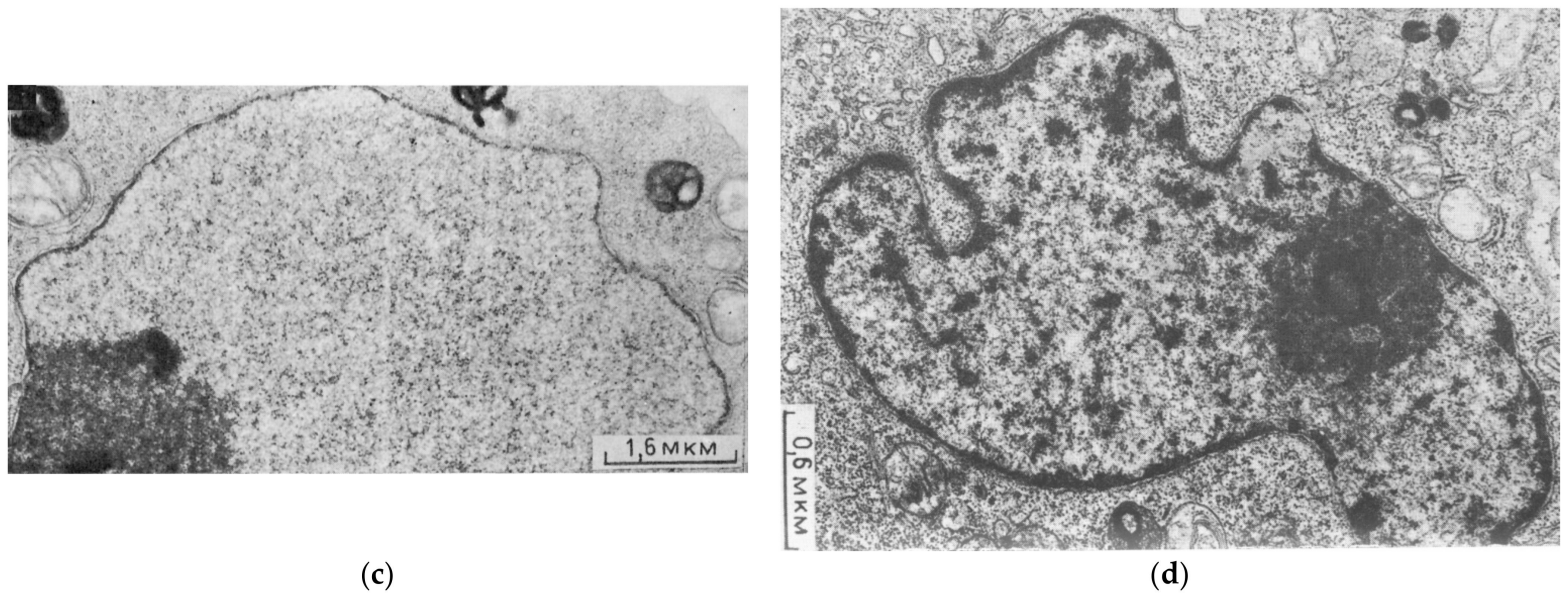
Segregation of the heterochromatin (HR) compartment in SV-40 transformed hamster fibroblast cell line 4/21: (**a**) the undifferentiated growth state; (**b**) after 2 min with bleomycin (01%/HBSS)—the pattern of HR nucleation; (**c**) 20 min bleomycin treatment—no compartmentation after deep chromatin fragmentation; (**d**) 1% DMSO 5-day-induced typical differentiation pattern of HR distribution in cell nuclei of the same cell line; (**a**–**d**) republished from [53].

The precipitation of the inactive domain forming heterochromatin network is in fact a phase transition. This can be appreciated, e.g., from experiments using the SV-40 transformed hamster cell fibroblast cell line 4/21 presented in Figure 5. In the logarithmic growth phase, the cells show a small amount of condensed chromatin at the nuclear envelope and the nucleolus, while the central nuclear space is nearly homogenous (Figure 5a). However, only 1–2 min of treatment with 0.1% bleomycin in the balanced Hanks salt solution (BHSS) is sufficient to change the pattern—the heterochromatin floccules dashing the radial paths from the nucleolus to the nuclear envelope and delineating the latter become conspicuously nucleated (Figure 5b). Deep DNA fragmentation with bleomycin (20 min) erased any chromatin compartmentation (Figure 5c). In turn, when this cell line was treated for five days with 1% DMCO, it accumulated DNA single-strand breaks and underwent differentiation with the typically condensed heterochromatin in the cell nuclei [26,53] (Figure 5d). As suggested by Sjakste, the terminal differentiation may be induced by DNA strand breaks themselves [87,88,89]. 

Interestingly, the precipitation of the heterochromatin network using cell imprints on microscopic slides with results presented in Figure 1c,d and Figure 3d was achieved after short (over 20 s) air-drying before fixation. This drying acts like mild osmotic stress and is reversible by wetting the cells [53]. In line with these observations, the biophysical study by [90] suggests that the increased recognition energy of ordered heterochromatic nucleosome arrays more densely packed near CTCF binding sites may favor the tendency to form stem structures (the sites of crystallization?). They are facilitated by the general osmotic stress and confinement pressure in the cell nucleus as well as by the additional pressure of DNA supercoiling acting near CTCF sites due to cohesion molecular motors, which actively form chromatin loops at these sites [91]. In addition, different polymer models have widely shown that non-equilibrated knotting and linking probability rapidly grows upon confinement [92].

Therefore, the same result achieved by air-drying and by localization microscopy and mathematical operations on the recorded data set of the freshly fixed cells corroborates that heterochromatin networks with certain topology and determinants in nucleus organization are predestined by the “physics of this compartment”. How are the CHR network determinants set in embryonic development? 

In mouse embryogenesis, lamina-associated domains (LADs) are settled in the zygote prior to topologically associating domains (TADs) [93]. The latter are conserved in evolution [94,95]. The link to lamina determines the variable chromatin landscape of mature cells [96] and the construction of the conventional or inverted (in retina rods of nocturnal animals) structure of cell nuclei [21]. However, at the two-cell mouse embryo stage, nucleolar precursors play a crucial role in generating a repressive environment for major satellite repeats, and this cannot be substituted for normal development by the landscape at the nuclear periphery [97]. It is also interesting to cite the studies from Sharakhov’s lab using a repetitive hybridization-based oligopaint approach on mosquito polytene cell nuclei, showing that the number of interchromosomal and the percentage of chromosome–nuclear envelope contacts were conserved among the species within the same cell type. They are moderately inversely related to interchromosomal contacts (r = −0.50, *p* = 0.012) [98]. Similarly, Ulianov et al. [99] showed on *Drosophila* cells that attachment of TADs to the nuclear lamina makes them more condensed but decreases overall chromatin density in the nucleus by stretching interphase chromosomes.

All in all, we can conclude that the intra-supra-chromosomal heterochromatin network seems to be both physically and developmentally preprogrammed and possesses two nuclear determinants: the nucleolus and nuclear lamina. Its general shape can be also revealed by perturbations depending in particular on the viscosity, charge, and humidity of both heterochromatin and active domains and DNA strand breaks. It changes function according to the laws of self-organization of viscous fluids [100,101] and also, possibly, emergent solid-like state [27].

## 5. The Radial-Concentric Order of PADs Networks, Their Relationship with Nucleoli and Nuclear Speckles

**Figure 6:**
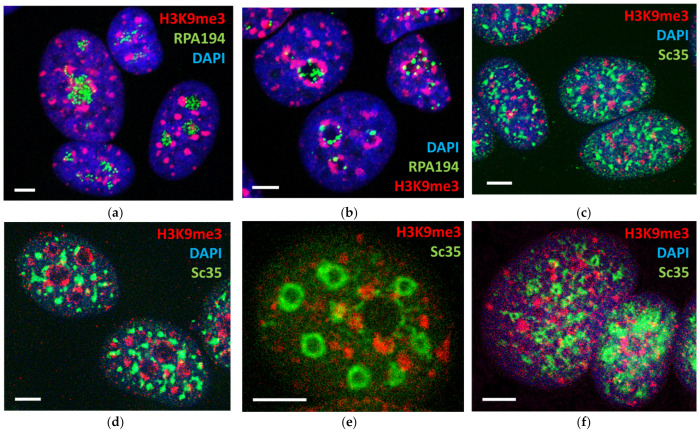
Partial inhibition of rRNA and mRNA synthesis by AcD and alpha-amanitin revealed the hidden radial-concentric spatial relationship between centers of nucleolar synthesis, perinucleolar PADs, and speckles; this order was destroyed after full suppression of transcription: (**a**) Control: the granules of RPA1 fill the nucleoli, indicating active rRNA synthesis; (**b**) AcD 0.2 µM/mL, 1 h—RPA1 form rare fused granules, indicating suppressed rRNA synthesis; H3K9me3 PADs compact and encircle the nucleoli; (**c**) control: elongated speckles and PADs seem disorderly distributed in the cell nucleus; (**d**) AcD 0.2 µM/mL, 1 h—clumped speckles surround the compacted shells of perinucleolar PADs; (**e**) alpha-amanitin 2 µM/mL for 2 h suppressing Pol II—an example of swollen empty speckles radially circumventing the deteriorating perinucleolar ring of PADs; (**f**) AcD 2 µM/mL for 5 h suppressing both RNA syntheses with chaotically distributed disarranged PADs and empty speckles. Scale bars = 5 µm.

**Figure 7:**
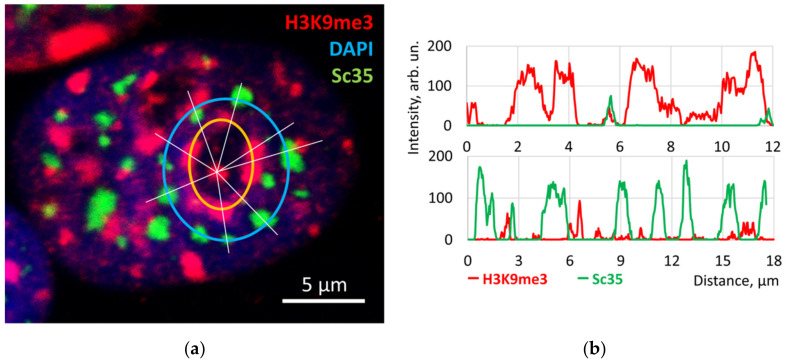

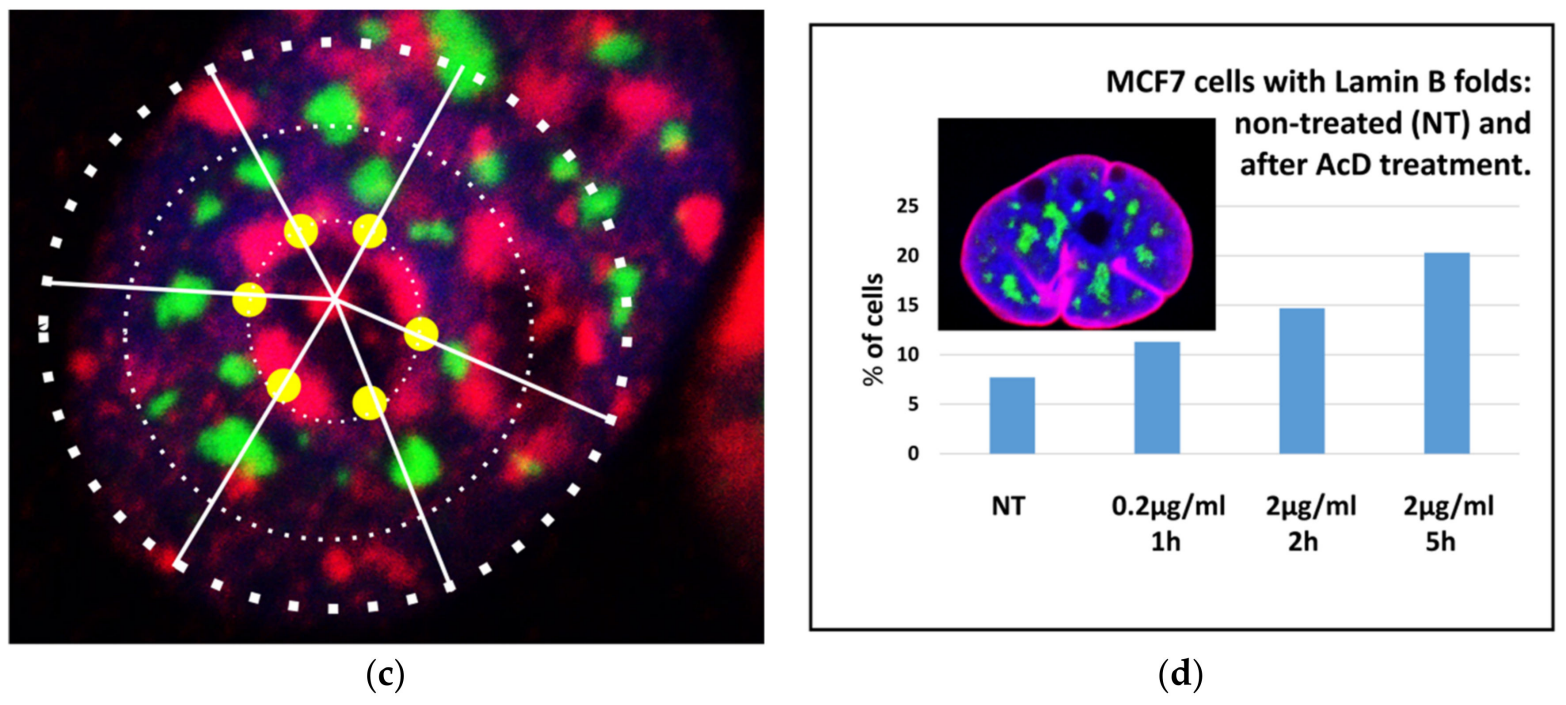
The radial-concentric nuclear order of the perinucleolar compacted PADs and speckles around them provoked in the course of transcription suppression by AcD treatment accompanied by gradual loss of lamin B stiffness: (**a**) After 1 h of AcD 0.2 µM/mL, a radial-concentric order of compacted perinucleolar PADs and the outer ring of compacted speckles was demonstrated in (**b**) by IF intensity measurements. (**c**) The radial-concentric model of the route-relay of the products of both rDNA and mRNA synthesis and splicing towards an arbitrary nuclear envelope (dashed). (**d**) Softening of nuclear lamin (evidenced by IF staining of lamin B1 (red)) testified as an increase of the proportion of cells with intranuclear lamin folds in the time course of suppression of RNA synthesis. Modified from [107].

Experiments with MCF7 cells inducing suppression of rRNA and mRNA synthesis at a smaller and larger concentration in the time course of actinomycin (AcD) treatment, and after alpha-amanitin suppression of mRNA and 5S RNA synthesis, were performed. To evaluate rRNA synthesis, the cells were stained by immunofluorescence for the cofactor of Pol I—RPA and fibrillarin involved in the processing of rRNA. They were also labelled for spliceosomes (speckles) by Sc35 [51] and for repressive PADs by anti-H3K9me3 antibodies. After 1 h of low AcD, fibrillarin concentration was diminished, the small RPA granules fulfilling the nucleoli in control (Figure 6a) fused in a few large granules retained at the nucleolar border (Figure 6b) before their full disappearance afterwards. In the earlier position, they were intermittent with H3K9me3 near-nucleolar large PADs (NORs) which condensed and coalesced onto the nucleolar margin (Figure 6b and Figure 7a). The elongated multiple speckles, which in control seemed disorderly scattered in cell nuclei (Figure 6c), also clustered after low AcD circumventing the collar of compacted nucleolar PADs (Figure 6d); both rings revealed a radial-concentric order (Figure 7a,b), which was not readily apparent in control. The action of a-amanitin suppressing Pol II and Pol III caused emptying and swelling of speckles, converting them into ring-like structures likely merging with PML bodies [102], which in some cells still revealed a concentric order (Figure 6e). In support, the reported movement and fusion of three or four spliceosomes under the suppression of RNA synthesis [103] should be mentioned. Coalescence of speckles upon suppression of RNA synthesis may be also favored by their phospholipid component participating in pre-mRNA splicing [103,104]. However, ultimately prolonged AcD or alfa-amanitin treatment disarranged the radial-concentric order initially induced by AcD (Figure 6f), and the topology of the studied elements again seemed nearly as random as in untreated cells (Figure 6c). Similarly, Haaf and Ward [105] showed that inhibition of RNA polymerase II transcription causes chromatin dispersion and loss of nucleolar structure.

The radial-concentric order of the nuclear components also includes the concentric ring of the nuclear envelope (NE) schematized as a dashed circle in Figure 7c. It is well-known that the attachment of heterochromatin to NE is dependent on lamins, particularly lamin B and its receptor [106]. We paid attention to the fact that in the time course of AcD action, the rigidity of the nuclear lamina decreased nearly three-fold as judged by a three-fold increase of cell proportion, clearly showing lamin B1-stained invaginations [107] (Figure 7d). Therefore, the situation after prolonged AcD action is a proxy to the loss of lamin B which is causing the redistribution of heterochromatin inside cell nuclei in some natural or experimental conditions [21]. Girard and colleagues [23] showed that loss of this association leads to loss of the bending stiffness of the lamina-associated heterochromatin.

## 6. Flexible Heterochromatin, Transcription Regulation, and Related Mechanobiology of the Actomyosin Network

**Figure 8:**
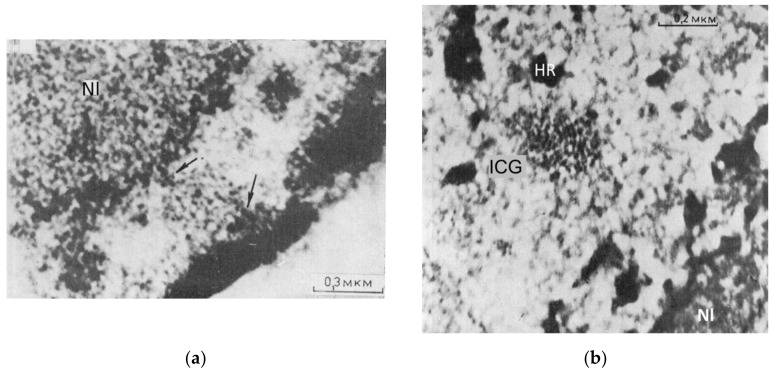

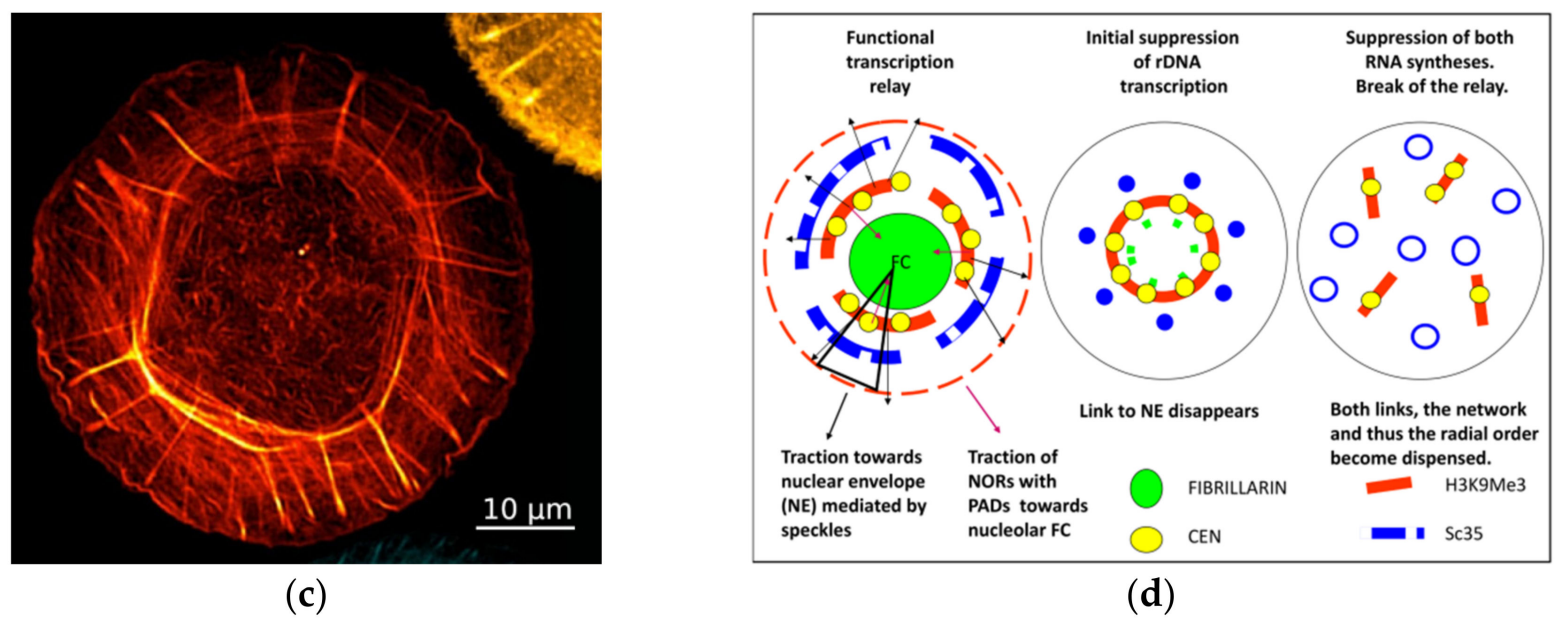
The speckles acting as radial spring pumps anchored between the nucleolar and perinuclear flexibly rigid heterochromatin shells and equipped by elastic actomyosin elements integrate the radial-concentric nuclear order for transcription pulsing. (**a**,**b**) EM thin sections of rat ascitic Zajdela hepatoma cells: (**a**) The links of the speckle (ICG) to the perinucleolar and perinuclear heterochromatin (arrows), Nl—nucleolus; (**b**) the view of the radial-concentric speckle substructure as revealed on shortly cold-PF-prefixed cells after short DNAse I treatment with 4.5 mM MgCl_2_ in phosphate buffer/sucrose before dehydration and epoxy-embedment (conventional contrasting). ICG—speckle, HR—heterochromatin, Nl—nucleolus. Republished from [53]. (**c**) The representative image of the circular-radial self-organization of F-actin filaments (stained by phalloidin) of human foreskin fibroblasts, structured soon after seeding on isotropic substrate; from [108]. (**d**) Schematic of the functional transcriptional relay in its relationship with speckles and the concentric rings of the nucleolar and perinuclear heterochromatin shells, deduced from experiments with suppression of RNA synthesis and the literature on the participation of the nuclear cytoskeleton in it. Modified from [107].

To further analyze this aspect, we shall touch on two points: the mechanobiology of transcription and transcriptional pulsing itself. EM studies of speckles (also called interchromatin granule clusters) examined in very active cancer cells revealed their direct link to both perinucleolar and perinuclear heterochromatin (Figure 8a), which thus exposed the order revealed by the AcD model. Interestingly, we could also reveal the radial-concentric pattern of the speckles themselves in the EM preparations shortly pretreated with DNAse I on prefixed material (Figure 8b). AcD provoked the radial-concentric organization of the main components involved in transcription machinery, with heterochromatin disclosing a spatial link between rRNA and mRNA synthesis and processing events. This may serve as a material base of transcriptional bursting through structured pulsations of the heterochromatin network premised in this review. The situation with low AcD mimics a return phase of transcriptional pulsing. To better explain these observations, we need to illuminate the link between CHR and transcription pulsing.

An actomyosin-based cytoskeleton using ATP energy plays a role in heterochromatin segregation and the organization of specific heterochromatin compartments: β-actin deficient cells exhibit changes in the spatial organization of H3K9Me3/HP1α—positive heterochromatin [109]. At the same time, one of the primary functions of the elastic nuclear actomyosin network is to maintain a chromatin landscape compatible with transcription [109,110,111]. How are these two functions coupled through the putative nuclear heterochromatin network? Elasticity and stiffness are the main biomechanical properties of the cytoskeleton network [112]. Additionally, flexible-bending rigidity and stickiness are the features of heterochromatin [23,90,113]. This would be a prerequisite for the cooperation of the two networks in the regulation of transcriptional pulsing, which is likely started from the nucleolus.

The involvement of fibrillar nuclear actin (F-actin) in the activation and process of transcription with all three RNA polymerases is well-established [110,114,115,116,117]. Upon serum stimulation of transcription, F-actin is seen accumulating in the nucleolus, transcription fabrics, and at the nuclear envelope [118] outlining the route of the transcription conveyor. Nuclear actin and the motor of actin, nuclear myosin (NM1), are required for RNA polymerase I transcription of rRNA [116,119]. Under AcD action, NM1 is transferred into the nucleolus [120], likely favoring the contraction of NORs. 

Actin is part of pre-initiation complexes and is necessary for transcription by RNA polymerase II; it is involved both in the initiation and elongation of transcription, acting through ribonucleoprotein interactions and the chromatin remodeling, but also interacting with pure DNA [121,122]. Nuclear actin is associated with a specific subset of hnRNP A/B-type proteins [123,124]. Actin is also needed for pre-mRNA splicing and its processing in speckles [125]. NM1-F-actin polymerizing complex activating Pol II needs the binding of bipolar phosphatidylinositol 4,5-bisphosphate and adapting by its disordered protein domain, which also enables liquid phase separation of nuclear speckles [126]. The role of speckles in the amplification of gene expression was recently stressed [127]. 

The involvement in RNA transcription of actomyosin activity of nuclear speckles, their phase separation, their anchoring to the perinucleolar and perinuclear heterochromatin, as well as their spring-like morphology, visualized by EM (Figure 8a,b), is apparent. It may assign the speckles enclosed in the alveoli of the heterochromatin network, a pump-spring feature involving this system in transcriptional pulsing. NM1 interacts with rRNA transcription initiation factor TIF-IA, which brings actin and NM1 in close proximity with each other. rRNA synthesis is regulated and responds to changes in the cellular environment, and TIF-IA has a central role in this regulation [116,119]. It thus provides feedback with the nucleolus sensing the cell environment, starting transcription pulsing aided by actomyosin. These studies show that the regulation of transcription is structured. At the same time, and it is most surprising, the process itself creates the assisting nucleoskeleton dynamic framework in *statu nascendi*. This network is constrained by the flexibly stiff heterochromatin attached to two nuclear boundaries, and similar circular-radial boundaries also determine a similar organization principle of the isotropic cytoplasmic F-actin network, as exemplified in Figure 8c [108]. 

The results of our AcD experiments together with the literature data analyzed above are deduced in the schematics in Figure 8d. It presumes that radial nuclear order is much dependent on the speckle springy traction of the nucleolar and nuclear RNA synthesis conveyor between the perinucleolar and perinuclear heterochromatin stiffly bending concentric rings. Release of the connection to nuclear lamin contracts the system inside the cell nucleus and exposes its radial-concentric character. When nuclear synthesis has been fully suppressed, both anchors of speckles to the perinucleolar and perinuclear constraints are released and the radial-concentric order is dispensed. Thus, nuclear speckles, postulated as a structure integrating the whole process of transcription [128,129], seem indeed capable of executing it through transcription pulsing of the heterochromatin network united with the actomyosin network which, in its turn, also follows and supports its radial-concentric character. The transcriptional pulsing seems to start from the nucleolus. Therefore, next we provide an insight into nucleolar pulsing and nucleolus-associated chromatin domains.

## 7. Nucleolar Pulsing and Nucleolus-Associated Domains (NADs)

Balbiani was the first who discovered vacuoles and their discharge from the nucleolus in active cells and called the nucleolus the “heart of the cell” [130]. Nucleolar pulsing was also described in plants, associated with transcriptional activity [131,132] and in mouse oocytes with particularly high synthesis of rRNA [133]. Pulsation of nucleolar rRNA synthesis was also revealed using confocal Raman microspectroscopy [134], and pulsation and coalescence of human nucleoli were registered by [135]. Hausnerova and colleagues [136] “report on the characterization of an RNA polymerase II transgene that is transcribed in the nucleolus. Using the MS2-GFP reporter system and live-cell imaging, we found transcription occurs on average for periods of 20 min. These ON periods alternate with periods of inactivity which last on average 29 min”. The authors suggest that observation of discontinuous transcriptional activity in the nucleolus may reflect cycling in the assembly and disassembly of active chromatin structure in and/or around rDNA genes.

In this connection, the question arises whether the emergence of function in transcription alveoli may be associated with nucleolus-associated domains (NADs). rDNAs are the most abundant genes, and the stability of the rDNA cluster was considered a key element of genome maintenance [137].

**Figure 9:**
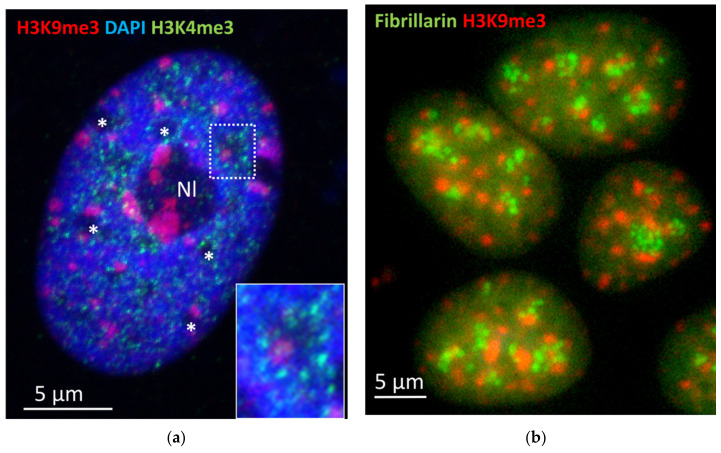
The topological relationship between PADs, NADs, and transcription hubs in MCF7 cells: (**a**) H3K9me3-labelled PADs are located near or inside the DAPI-poor alveoli decorated by H3K4me3-positive foci (enlarged on insert) which are (**b**) often occupied with the fibrillarin-marked nucleoli.

NADs are defined as genomic regions enriched at the nucleolar periphery in interphase cells [73]. In addition to the nucleolar organizers (NORs) in five acrocentric chromosome pairs of the human genome [138], when using deep sequencing of chromatin associated with biochemically purified nucleoli in Hi-C and other labelling methods, it was revealed that specific chromatin domains from most human chromosomes may associate with nucleoli [139]. The NADs (from 0.1 to 10 Mb in length, median at 749 kb) comprising 38% of the human genome contain rDNA sequences and H3K9me3-enriched heterochromatin associated with a-satellite DNA and centromeres [140]. They are mainly heterochromatic and correlate with late replicating loci. Genome-wide mapping showed that interactions of NADs control the organization of interphase chromosomes in the 10–50 Mb distance range [71,140]. The association of large ~2 µm (average) rosettes in the heparin-treated nuclear network with nucleoli was highlighted in their first description [52]. It is worth noting that EM shows that these alveolus-rosettes are organized around a cluster of heterochromatin often seen intruded in it with the “foot” (Figure 1b). It is a characteristic feature of NORs [141], also seen in deconvoluted AO-DNA staining (Figure 1c). Intriguingly, NADs partly overlap with LADs [142]. The association of NADs with nucleoli is still largely preserved in cellular senescence despite massive nuclear reorganization [71]. However, NAD regions unique to either young or senescent cells were found enriched in protein-coding genes. Moreover, the loss of nucleolar association in either young or senescent cells correlated with higher gene expression; conversely, the gain of NAD status correlated with decreased gene expression. Thus, most data define the nucleolar periphery as a silencing hub that shows the most stable associations in the genome, appears first in embryogenesis, and serves as a docking and chromatin mobility limiting site [73,143]. These results are in accord with our observations on the radial vector from the nucleolus and back in transcriptionally active and inactive cells, respectively, in chicken embryonal chondrocytes, as described above. The most recent data on NADs in mouse embryonic stem cells and fibroblasts, besides a large NADs subset overlapping with LADs, revealed a subset of smaller NADs free from binding to nuclear lamin—corresponding more dynamic chromocenters with higher gene density, including developmental and differentiation genes [144,145]. The authors suggested that specific trans-acting factors are required for such localizations (which, in our view, is equivalent to the recognition of the network of these dynamic NADs). The topological proximity of PADs with the nucleoli in the DAPI-poor nuclear spaces decorated by H3K4me3-foci can be seen in Figure 9a,b. It cannot be excluded that just a portion of dynamic NADs/PADs is associated with scale-free dynamics reported in our recent work on the MCF7 model cell system [44]. The enrichment of NADs with centromere repeats may be also mediated by centromere RNA [146]. Nucleoli in G1 cells are very dynamic—fusing and splitting; the reiterated ribosomal DNA sequences are also easily amplified and highly variable in size [135,146,147], flexibly adapting to cell needs, and are the first undergoing replication stress during genome instability and cell senescence [148].

In any case, the available molecular and morphological data indicate that most NADs are associated with PADs, and this link can be hypothesized to play a role in the formation of functional alveoli and scale-free transcriptional pulsing of their mutual network coordinating rRNA and mRNA synthesis in the radially directed relay. Therefore, we should shortly review some data on transcriptional pulsing.

## 8. Transcriptional Pulsing and Synchronization in a Cell Population

Transcriptional bursting, also known as transcriptional pulsing, is a fundamental property of genes, which have been observed in diverse organisms, from bacteria to mammals [149]. The burst frequency is primarily encoded in enhancers, and burst size in core promoters, while cell type-specific gene expression is primarily shaped by changes in burst frequencies [12]. Cultured cells display cell cycle-independent collective dynamics of gene transcription fluctuations with a period of 20–40 min [9]. The stress response of Zaidela ascites carcinoma cells (washed and incubated in PBS in a plugged vial), seen as a few ~90 min collective waves of mRNA synthesis increased up to two to three times, was registered along with a bi-stable switch for the increased superhelicity of DNA opposed by the emergence and subsequent repair of DNA single-strand-breaks; these oscillations further faded and led to cell death [13,150]. MCF7 cells synchronized with the inhibitor interrupting mRNA elongation were recently examined by 4sUDRB-sequencing for genome-wide transcription [151]. The collective burst of 11,644 genes (each varying in intensity) was registered between 5 and 20 min, with a lower phase lasting until 40–50 min. Interestingly, the bursting period is similar to that described by [136] for nucleolar pulsing. Similar periodicity was also described for the pulsing of large chromocenters in phenobarbital-stressed rat hepatocytes [152]. In general, these results are consistent with our hypothesis on the coordinated pulsing of the PAD-NAD scale-free network involving the nucleoli. 

The sense of radial-circular nuclear organization for transcriptional pulsing is not only in the structural conveyor coordinating the production of pre-ribosomes with the synthesis and maturation of mRNA but also in the adaptation of this process to the needs of the cell as an open system. It means feedback from sensing the environment and cell community which, in case of life threat (and likely, in reprogramming), can become synchronised. In other words—it is an element of explorative adaptation requiring the system to act as a whole. As described above, the cytoskeleton is part of this pulsing-sensing feedback where the assembly and interactions between DNA and F-actin and phase separation functions are also a subject of the self-organizing forces [121]. But the question remains, how does this dynamicity fit with the robust regulation of gene expression in tissue differentiation [153] and, ultimately, how is the tissue-specific code established and read?

## 9. Replication Timing and the Tissue-Specific Heterochromatin Code as Address in a Coordinate System

**Figure 10:**
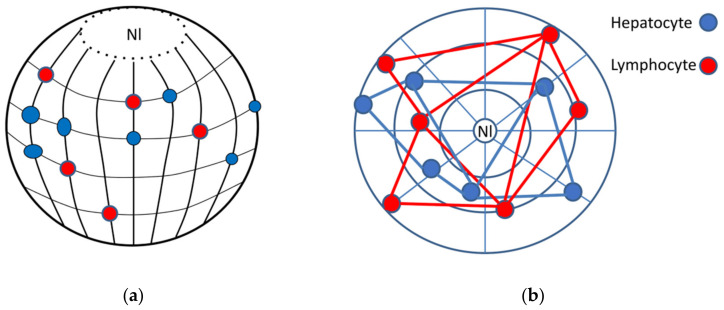
Replication timing and the tissue-specific “heterochromatin code” in a coordinate system for two different cell types (arbitrary hepatocytes and lymphocytes). (**a**) Hypothesis: The tissue-specific positional information is created by the latitude positions of silencing constitutive heterochromatin (CHR) clusters set (designed as red and blue balls) by replication timing upon the radial chromosome longitudes as the “3–4 D address” (to be specified by topology-associated domains (TADs)). (**b**) Principle sketch of the CHR clusters positions in the cell nucleus in a radial-concentric “geographic map” of coordinates (on 2D image) determined by Delaunay triangles. Nl—nucleolus.

We can suggest that the tissue-specific code is established by genome-wide stable replication timing (earlier for active genes and later for inactive compartments, with constitutive heterochromatin being mostly the latest [154,155], when it is applied to the radial arrangement of chromosomes in the late S-G2 phase (schematically presented in Figure 10a). It is noteworthy that both replication timing and transcriptional pulsing likely have a radial-circular chromatin network organization as material substrate. The same principal organization has been found for the F-actin cytoskeleton of mammalian cells. Being involved in transcription, the latter should enable the elastic transcriptional pulsations. Replication timing information is concealed by corresponding epigenetic histone modifications for particular nucleosome conformation states, which are known as setting the same chromatin marks of the DNA segments replicating at the same time [154,156]. This enables their spatial assembly by nucleosome conformation complementarity in interphase [157,158]. The epigenetic histone setting may provide the epigenetic memory that is stable through the cell cycles [159,160]. Based on our findings and literature analysis in this review, we pose that a “tissue-specific 3D address” of positional information can be obtained from the topological and physical characteristics of heterochromatin (“heterochromatin code”) set in a system of coordinates. The idea is somewhat close to the “unified matrix hypothesis” of tissue differentiation by Klaus Scherrer [35]. We also speculate that transcription bursting adjusts the pulsing frequency and amplitude of the conserved elements specifying this code—namely TADs—to this tissue-specific heterochromatin code of replication timing, and that bivalent chromatin can serve as an adaptor.

“Heterochromatin code” is thus tissue-specific positional information created by the latitude positions of silencing constitutive heterochromatin clusters set by replication timing upon the radial chromosome longitudes (an analogue of chromosome banding). It can be mathematically determined using segmentation of the cell nucleus image in a radial-concentric matrix around a central origin, e.g., a center of the nucleolus. Inside this matrix, the “geographic” map locating the coordinates of constitutive heterochromatin clusters can be defined (schematically shown in Figure 10b). Mathematical operations, for instance, radial pair correlation functions, edge length codes after Delaunay triangulation, or operations of algebraic topology determining invariants to distinguish different topological spaces, can be applied. They allow the comparison of different states as mentioned in Section 1 in its simplest binary form “active”–“non-active”. A more complex parameter set of the heterochromatin code could also include more selective substages.

## 10. Conclusions

Guided by a mesoscopic approach to complex systems, we attempted to unveil the presence of “organization principles” from the correlation structure of the studied phenomenon, where “all the entities can be considered as networks of interacting parts” [47]. Thus, we applied an empirical approach for the revelation of the constitutive heterochromatin network showing similar features in different animals and cell types. We mostly used the methods of microscopic image analysis in different functional situations and put this data in the context of physical power-law regulation. We also used the modern nucleome update, using molecular methodology indicating the leading role of heterochromatin contacts for maintenance of nuclear organization. We were also guided by the understanding of complex dissipative systems as undergoing structured pulsations for the feedback adaptation to the environment and signaling from the cell community. We also posed the most difficult question of how the genome can be regulated by heterochromatin as a whole for robust reading of genetic information in differentiated tissues. Here, we applied the paradigm of position information presaged by Schrödinger [2] and introduced in a coordinate system for morphogenesis by Wolpert [48]. All analyzed data converge to the radial-concentric organization principle of the cell nucleus that sets epigenetic information into the radial position of chromosomes through replication timing and executes it in transcriptional pulsing. This instructs protein synthesis in a tissue-specific manner and gets permissive feedback from the environment, in particular with the aid of a similarly organized elastic actomyosin network which emerges together with a transcription conveyor. We have underlined the scale-free bursting of the heterochromatin network, along with pulsing of transcriptional hubs enclosed in it, and the constraint of the repressive nucleolar heterochromatin for the vectorial regulation of these processes. Thus, the dynamic supra-chromosomal network allows for the integration of incoming microenvironment signals and for the genome to act as a whole.

## Figures and Tables

**Table 1:** The average diameter of the heterochromatin network alveoli was determined by five different methods in chicken, rat and human cells.

Species	Cell Type	Method of Heterochromatin Network Discrimination and Alveoli Diameter Determination	Diameter of Alveoli (µm)	Source
Rat	Thymocytes	Treatment with heparin of isolated nuclei on EM supports; interactively determined	~2	[52]
Human	Breast cancer line SkBr3	H3K9me3, localization microscopy; semi-automated	1.7 ± 0.4	own data, unpublished
Human	Breast cancer line SkBr3 (30 min post 4 Gy)	H3K9me3, localization microscopy; semi-automated	1.9 ± 0.5	own data, unpublished
Chicken	Embryonal chondrocytes	Specific Feulgen-type DNA staining of cell imprints; mathematic skeletonization	1.8 ± 0.7	[57]
Human	Breast cancer line MCF7	Acridine orange DNA staining (after RNAse); epifluorescence, deconvolution, image analysis	1.87 ± 0.74	current
Human	Breast cancer line MCF7 (AcD 2 µg/mL, 5 h)	Acridine orange DNA staining (after RNAse); epifluorescence, deconvolution, image analysis	2.12 ± 0.67	current
Human	Breast cancer line MCF7	Affinity stain for heterochromatin with toluidine blue; image analysis	1.7 ± 0.4	current
